# Retracted: Clinical Brain Death with False Positive Radionuclide Cerebral Perfusion Scans

**DOI:** 10.1155/2016/2057806

**Published:** 2016-03-15

**Authors:** Case Reports in Critical Care

The article titled “Clinical Brain Death with False Positive Radionuclide Cerebral Perfusion Scans” [1] has been retracted as the scans in Case 2 of the article were interpreted incorrectly. As a result, the conclusions are not reliable.
